# Action of lytic polysaccharide monooxygenase on plant tissue is governed by cellular type

**DOI:** 10.1038/s41598-017-17938-2

**Published:** 2017-12-19

**Authors:** Brigitte Chabbert, Anouck Habrant, Mickaël Herbaut, Laurence Foulon, Véronique Aguié-Béghin, Sona Garajova, Sacha Grisel, Chloé Bennati-Granier, Isabelle Gimbert-Herpoël, Frédéric Jamme, Matthieu Réfrégiers, Christophe Sandt, Jean-Guy Berrin, Gabriel Paës

**Affiliations:** 1 FARE Laboratory, INRA, University of Reims Champagne-Ardenne, 51100 Reims, France; 2 BBF, INRA, Aix Marseille University, Polytech’Marseille, 13288 Marseille, France; 3 Synchrotron SOLEIL, 91190 Saint Aubin, France

## Abstract

Lignocellulosic biomass bioconversion is hampered by the structural and chemical complexity of the network created by cellulose, hemicellulose and lignin. Biological conversion of lignocellulose involves synergistic action of a large array of enzymes including the recently discovered lytic polysaccharide monooxygenases (LPMOs) that perform oxidative cleavage of cellulose. Using *in situ* imaging by synchrotron UV fluorescence, we have shown that the addition of AA9 LPMO (from *Podospora anserina)* to cellulases cocktail improves the progression of enzymes in delignified *Miscanthus x giganteus* as observed at tissular levels. *In situ* chemical monitoring of cell wall modifications performed by synchrotron infrared spectroscopy during enzymatic hydrolysis demonstrated that the boosting effect of the AA9 LPMO was dependent on the cellular type indicating contrasted recalcitrance levels in plant tissues. Our study provides a useful strategy for investigating enzyme dynamics and activity in plant cell wall to improve enzymatic cocktails aimed at expanding lignocelluloses biorefinery.

## Introduction

Plant cell walls constitute the largest renewable source of biomass on Earth that can supply environmental benefits for the production of fuel, chemicals and materials. They are composed by lignocellulose made from three main polymers (cellulose, hemicelluloses, lignin) assembling as a network whose structural and chemical complexity hampers hydrolysis of cellulose by enzymes and micro-organisms^1^. In addition, cell walls display high variability depending on genetic and environmental factors, as well as plant tissue and cell types^2–4^. Understanding plant cell wall complexity during lignocellulosic bioconversion is therefore important to identify critical features impacting hydrolysis, for optimising pretreatments of biomass^5^ and enzyme cocktails^6^.

Investigation of the dynamics of lignocellulose hydrolysis requires physicochemical characterization and multiscale visualization^7,8^. Combined approach using multiple microscopic techniques including scanning electron microscopy, atomic force microscopy, light microscopy, immunocytochemistry and microspectrometry can be used to monitor microstructural and topochemical heterogeneity of plant cell walls and their recalcitrance at tissue, cell and subcellular levels^9–15^. In addition, spatial and temporal imaging of enzymes distribution within lignocellulose substrates can be carried out by means of immunoprobe or fluorescent labelled protein^16–18^ to study accessibility at the molecular scale^19,20^. However real time imaging of cell wall microstructure and enzyme distribution during bioconversion still remains challenging.

Enzymatic degradation of cellulose and hemicelluloses involves several types of enzymes, namely glycoside hydrolases that work synergistically^21,22^. To overcome the recalcitrance of plant polysaccharides, filamentous fungi and bacteria secrete lytic polysaccharide monooxygenases (LPMOs) that perform oxidative cleavage of glycoside bonds^23–25^. In industrial processes, addition of LPMOs to cellulolytic cocktails leads to the reduction of the enzyme loading required for efficient saccharification of lignocellulosic biomass^26^. These powerful enzymes are copper-containing enzymes classified within the auxiliary activity (AA) class of the CAZy database (www.cazy.org ^27^) in AA9-AA11 and AA13 families. AA9 LPMOs are mostly active on cellulose but some have been shown to act on xyloglucan and glucomannan^25,28^. Despite their recognized boosting effect on biomass hydrolysis, AA9 LPMOs activity has been essentially investigated on model substrates with only sparse studies focusing on the insoluble fraction of the substrate that show their disruptive action at the surface of cellulosic fibers^29–31^. To our knowledge, no studies have investigated the impact of LPMOs on lignocellulose substrate at the plant tissue and cell wall scales.

In this study, we have followed the LPMO action on miscanthus as a model substrate using dynamical imaging. Enzymes from a commercial *Trichoderma reesei* cellulose-active cocktail (Celluclast^®^) were tested in combination with a fungal AA9 LPMO from *Podospora anserina* (*Pa*LPMO9E) bearing a family 1 carbohydrate- binding module and acting on cellulose only^32^. Taking advantage of the high tuneability, brilliance and stability of the synchrotron light source, deep-UV autofluorescence of the enzymes and of the cell walls were used to track the enzyme localization and to follow real-time cell wall modifications at early stages of enzymatic degradation. Synchrotron Fourier Transformed InfraRed (FTIR) microspectrometry enabled to follow *Pa*LPMO9E-mediated chemical changes of the cell walls. To this end appropriate miscanthus samples were prepared and experimental design was set-up to allowing real-time monitoring changes of selected tissues and cells during the action of enzymes under hydrated conditions and optimal temperature.

## Results and Discussion

### Enzymatic hydrolysis of miscanthus

**Figure 1:**
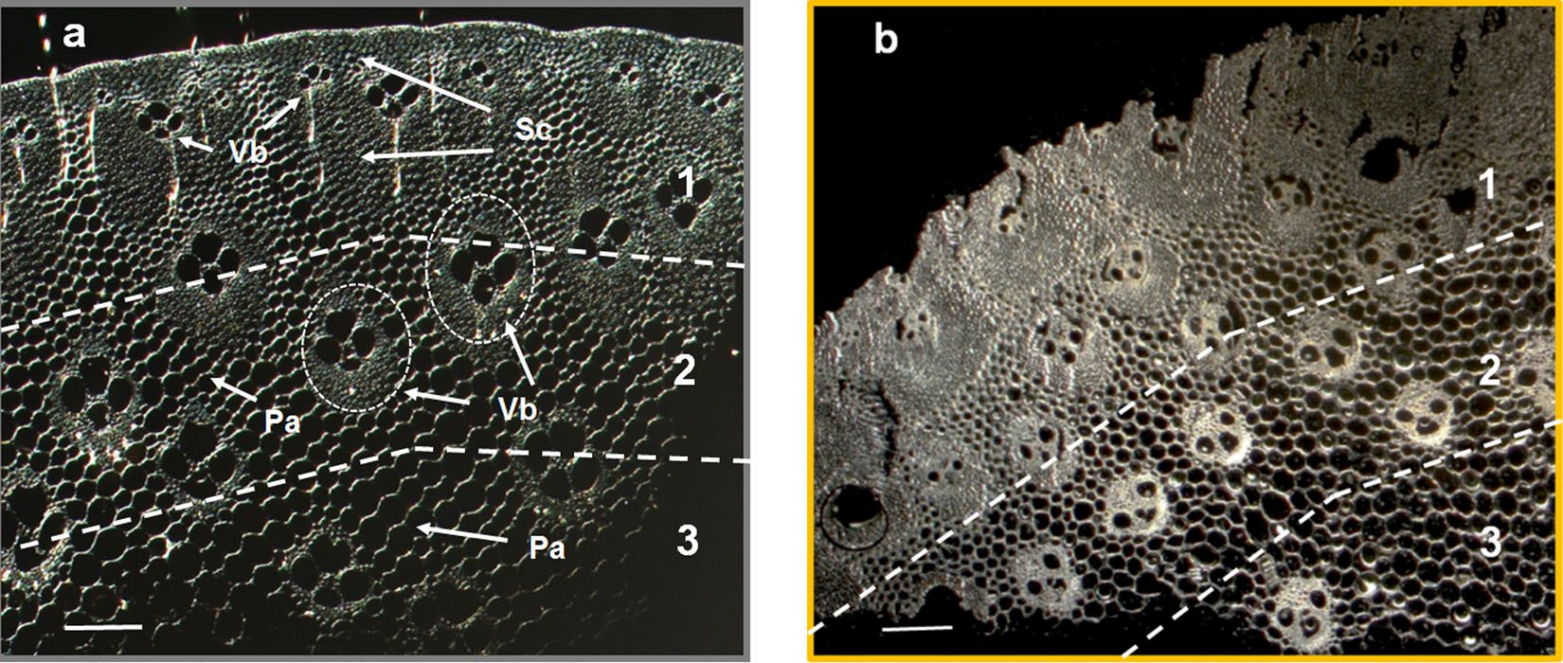
Visible light imaging of stem tissue architecture of untreated and chlorite-treated miscanthus sections. (**a**) Untreated miscanthus. (**b**) Chlorite-treated miscanthus. For each sample, transverse sections show three main regions: 1) rind with thick layer of sclerenchyma (Sc) and small vascular bundles (Vb), 2) middle region rich in interfascicular parenchyma (Pa) and vascular bundles which are surrounded by a wide sclerenchyma sheath, 3) innermost region rich in parenchyma. Scale bar is 200 µm.

Although LPMOs are known to boost the saccharification of plant cell walls^30^, they have never been tested on miscanthus, which is a candidate perennial bioenergy crop producing high biomass with low input and environmental impact^33–35^. The ability of *Pa*LPMO9E to improve saccharification of pretreated miscanthus was first evaluated on steam-exploded miscanthus. Indeed, steam explosion is currently among the most effective pretreatment for second generation biofuels production and can be scaled-up industrially^36–38^. Steam explosion consists in application of steam at a high pressure followed by rapid decompression, inducing destructuration of lignocellulose and tissue disruption with lignin recondensation (Supplementary Fig. 1a)^39,40^. Addition of *Pa*LPMO9E to Celluclast^®^ increased glucose yields by 87% (Supplementary Fig. 1b) in comparison to the action of Celluclast^®^ alone. *Pa*LPMO9E was previously shown to oxidatively cleave cellulose at the C1 position of the glucose unit^32^. Accordingly, C1-oxidized cello-oligosaccharides were detected following the addition of *Pa*LPMO9E alone or with Celluclast^®^ on steam-exploded miscanthus (Supplementary Fig. 1c), confirming the cleavage of cellulose chains by *Pa*LPMO9E. Thus the action of *Pa*LPMO9E was likely responsible for the improvement in glucose yield. Having demonstrated that *Pa*LPMO9E favoured cellulose enzymatic conversion of steam-exploded miscanthus, the boosting effect of *Pa*LPMO9E was investigated at the tissue and cell wall scales. Such imaging requires lower severity of pretreatment to make observations of undamaged tissues. The steam explosion pretreatment causes relocation of lignin and fragmentation of lignocellulose, which does not allow tracking the effect of *Pa*LPMO9E on specific tissues and cells forming miscanthus internode. Rather, sodium chlorite-acetic acid treatment (so-called chlorite-pretreatment) was applied to small portions of miscanthus stem samples to preserve cell and tissue organization. Although this reagent may induce different cell wall structure compared to steam explosion, it is widely used as delignification agent in paper industry for example^11,41,42^. Regarding chemical composition, chlorite pretreatment induced a strong decrease of the lignin content of miscanthus sample (from 26% to 5% dry matter) in comparison to untreated sample (Supplementary Fig. 2). Consequently, proportion of polysaccharides, especially cellulose, increased in chlorite-treated miscanthus (from 49 to 60% dry matter). Despite large variations in composition, the tissue and cell architecture of the sample was preserved as revealed by observations of transverse section of miscanthus stem at the cell scale (Fig. 1)^3,12^. Since stem miscanthus includes different cell types with typical organization pattern as comparable to grass species^43^, three main regions were distinguished from the outer to the inner part of a transverse section (Fig. 1a): i) Region 1: the rind mainly including thick layer of sclerenchyma, with a small amount of small vascular bundles; ii) Region 2: the middle region, rich in interfascicular parenchyma and vascular bundles which are surrounded by a wide sclerenchyma sheath; iii) Region 3: the innermost region rich in parenchyma. Although outermost cells were slightly altered by chlorite treatment, the whole stem tissue architecture was not altered by the treatment (Fig. 1b).

**Figure 2:**
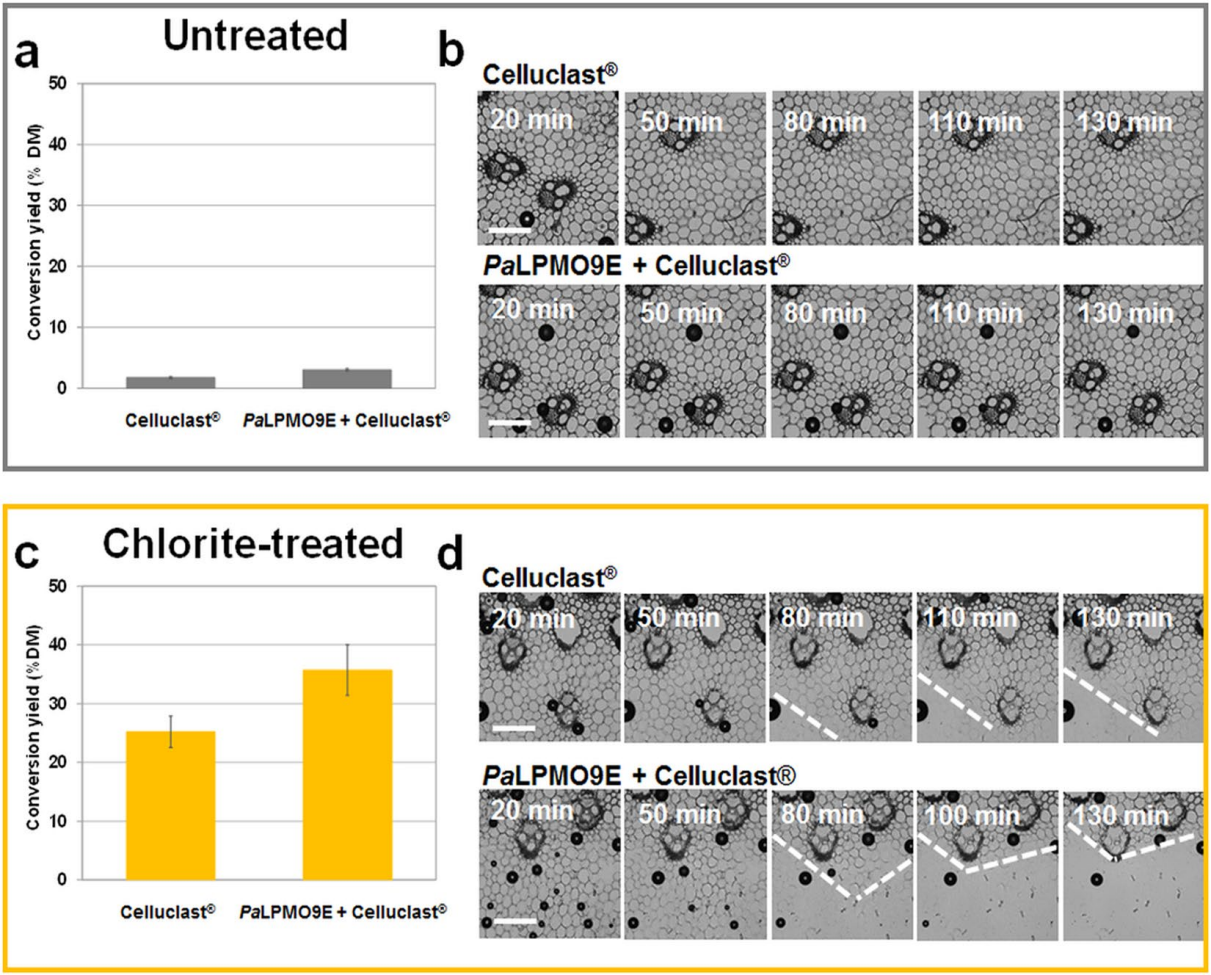
Effect of *Pa*LPMO9E on the degradation of untreated and chlorite-treated miscanthus. Enzymatic conversion of (**a**) untreated and (**c**) chlorite-treated miscanthus. Visible light time-lapse imaging of Region 2 of (**b**) untreated and (**d**) chlorite-treated miscanthus with Celluclast^®^ alone and *Pa*LPMO9E + Celluclast^®^. Conversion yields are given as equivalent glucose after 72 h incubation and expressed as % dry matter. Dotted lines delineate the degraded parenchyma area region. Scale bar is 200 µm.

Considering the untreated miscanthus, 72 hours incubation with Celluclast^®^ only permitted a limited conversion of miscanthus, which was slightly enhanced by the addition of *Pa*LPMO9E (Fig. 2a). The chlorite-treated sample conversion as determined by DNS method, was ~25% dry matter with the cellulase cocktail alone, and reached 38% upon addition of *Pa*LPMO9E (Fig. 2c) indicating a 50% improvement of enzymatic degradation. The same trend was observed when glucose was quantified by the glucose RTU kit method. Tissular changes were followed in the 3 regions defined in Fig. 1. For both untreated and chlorite samples, the Region 1 that contains thick-wall sclerenchyma was unaltered over a time-period of more than 2 hours when incubated with *Pa*LPMO9E + Celluclast^®^ except of phloem cells in vascular bundles (Supplementary Fig. 3). This result is in good agreement with previous microscopic observations of longitudinal sections of wheat straw showing recalcitrance and barrier effect of the outermost stem tissues^10^. In contrast, the Region 3 of chlorite–treated miscanthus showed fast degradation following addition of Celluclast^®^, which is in line with the low recalcitrance of pith parenchyma observed for transverse or longitudinal sections of pretreated grass samples^10,11^. Complete degradation of this region was reached within less than 2 hours making difficult real-time microscopic monitoring of the changes occurring on selected cells along enzymatic conversion. For untreated miscanthus, region 3 was almost unaltered after several hours incubation. In Region 2 no changes in tissular architecture of untreated miscanthus were observed after 2 hours of incubation with enzymes (Fig. 2b). In contrast, chlorite-treated miscanthus displayed significant modifications of Region 2 that mainly showed degradation of the parenchyma (Fig. 2d). More precisely, degradation occurred faster when the *Pa*LPMO9E was present since the parenchyma had disappeared after 80 min of incubation with *Pa*LPMO9E + Celluclast^®^ but was still present after 130 min with the cellulase cocktail alone. Overall, direct light imaging of enzymatic incubation of chlorite-treated miscanthus indicates tissue heterogeneity regarding susceptibility to enzymes. These observations suggest that the increase in the rate of enzymatic conversion of chlorite samples by addition of *Pa*LPMO9E seems to be explained by a higher susceptibility of parenchyma in Region 2, thereby revealing that synergistic effect of accessory enzymes on cell wall saccharification varies at tissular level.

### Synchrotron fluorescence imaging

**Figure 3:**
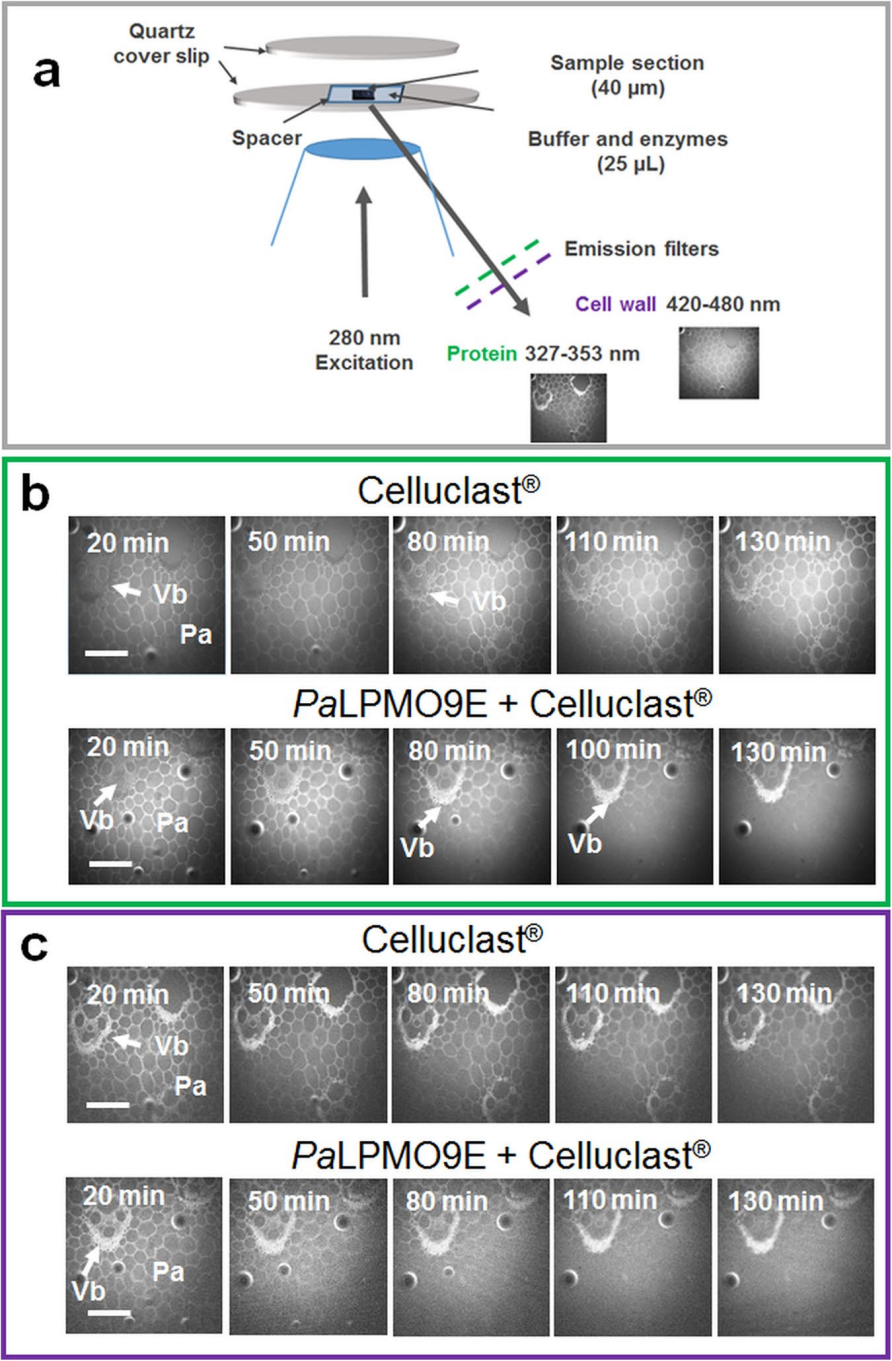
Fluorescence time-lapse imaging of enzymatic conversion of chlorite-treated miscanthus. (**a**) Experimental set-up of the deep-UV fluorescence imaging at synchrotron beamline. (**b**) Evolution of protein fluorescence during hydrolysis by Celluclast^®^ alone or with *Pa*LPMO9E. (**c**) Evolution of cell wall fluorescence during hydrolysis by Celluclast^®^ alone or with *Pa*LPMO9E. Scale bar is 200 µm.

To visualize both enzymes and cell wall during enzymatic saccharification, time lapse fluorescence imaging at different emission wavelengths was implemented (Fig. 3a). First, enzymes were followed through their aromatic amino acids (mainly tryptophan) using synchrotron UV fluorescence to track enzymes *in situ* (Fig. 3b)^44,45^. For the untreated miscanthus, fluorescence imaging of Region 2 did not indicate the presence of protein even after more than 2 hours incubation with the *Pa*LPMO9E + Celluclast^®^ (Supplementary Fig. 4). This observation is consistent with visible light imaging of miscanthus (Fig. 2b) indicating the absence of any structural modification in the same conditions. In contrast, for the chlorite-treated sample, protein fluorescence was detected in the cell walls and the increase in fluorescence was more important and occurred earlier in the presence of *Pa*LPMO9E (Fig. 3b). As a negative control, the same sample incubated in buffer without enzymes did not show any significant modifications (Supplementary Fig. 5). Importantly, sclerenchyma sheath of the vascular bundles displayed higher fluorescence intensity after parenchyma degradation (Fig. 3b) suggesting that enzyme distribution in these cells walls is favoured by combining *Pa*LPMO9E and Celluclast^®^ and may allow degradation of these cells. Observations of the protein distribution thus demonstrate a faster progression of the cellulases in the presence of *Pa*LPMO9E and a preferential cell wall degradation within stem cells. Following observation of proteins, cell walls were imaged by following the fluorescence of phenolic compounds using an excitation wavelength of 280 nm and an emission filter at 420–480 nm (Fig. 3a). In agreement with visible light observation and protein imaging, the cell wall remained unchanged for untreated miscanthus after 2 hours incubation with buffer or enzymes (Supplementary Fig. 4). In contrast, time lapse imaging of chlorite-treated miscanthus showed a decrease in the cell wall fluorescence of region 2 parenchyma during enzymatic saccharification (Fig. 3c), while no variation of fluorescence was observed when the sample was incubated in buffer alone (Supplementary Fig. 5). The decrease in fluorescence in the presence of *Pa*LPMO9E appeared higher than without (Fig. 3c), as observed previously with visible light imaging (Fig. 2d). Incubation of both untreated and chlorite samples with buffer even after 4 hours of incubation did not show any fluorescence change, indicating that the synchrotron beam light did not induce significant change during the course of the analysis (Supplementary Figs. 4 and 5). Overall fluorescence UV synchrotron imaging allowed to distinguish variations in dynamic of enzymes and in cell wall breakdown according to the type of cell and tissue.

### Synchrotron FTIR analysis

**Figure 4:**
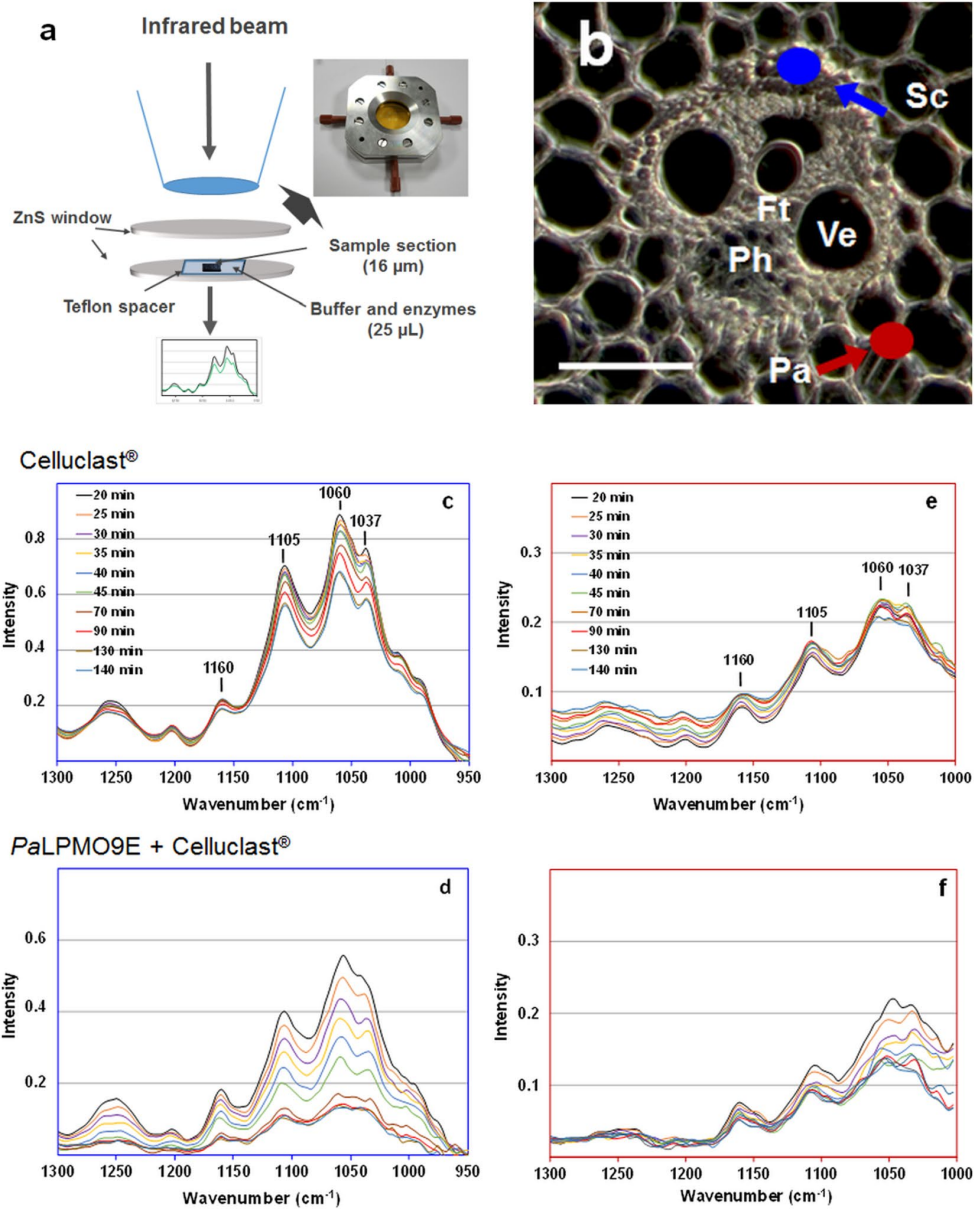
Real time monitoring of FTIR spectra of chlorite-treated miscanthus during enzymatic conversion. (**a**) Experimental setup using the microfluidic device. (**b**) Vascular bundles cells and localization of sclerenchyma (Sc) and parenchyma (Pa) (scale bar is 50 µm). Evolution of sclerenchyma FTIR spectra during hydrolysis (**c**) by Celluclast^®^ alone or (**d**) with *Pa*LPMO9E. Evolution of parenchyma FTIR spectra during hydrolysis (**e**) by Celluclast^®^ alone or (**f**) with *Pa*LPMO9E. Scale bar is 100 µm.

As for visible light and fluorescence imaging, real time FTIR characterization of miscanthus samples was achieved in a microfluidic device that allowed following structural changes of the cell walls in the presence of enzymes (Fig. 4a). More specifically, real time FTIR imaging was performed on Region 2: vascular bundles that include different cell types (sclerenchyma, tracheids, phloem) and adjacent parenchyma cells (Fig. 4b). As compared to untreated miscanthus, infrared spectra of the chlorite sample were altered at initial time of analysis (20 min): intensity of the bands assigned to lignin (1505 cm^−1^ and 1460 cm^−1^) decreased in agreement with compositional data (Supplementary Fig. 2). FTIR spectra did not change during the following 2 hours of incubation with buffer (Supplementary Fig. 6) showing the stability of the miscanthus section in the absence of enzyme. Enzymatic degradation of phloem cells were not monitored by FTIR imaging, as these thin-walled and unlignified cells were quickly degraded by Celluclast^®^ (less than 30 min). When incubated with enzymes, the spectra of the chlorite-treated miscanthus showed decreasing intensity of several vibration bands assigned to lignocellulosic polysaccharides (cellulose and hemicellulose) as illustrated by the corresponding spectral regions recorded for sclerenchyma and parenchyma (Fig. 4c–f). Compared to incubation with Celluclast^®^ (Fig. 4c,e), addition of *Pa*LPMO9E induced stronger alteration of the spectra (Fig. 4d,f). This indicates that the progressive degradation of the cell walls by cellulases is enhanced by adding *Pa*LPMO9E in agreement with previous fluorescence observations. Two main bands at 1060 and 1105 cm^−1^ representative of cellulose^13^ were selected to compare the course of the degradation at the cell level (sclerenchyma, parenchyma, tracheids). Intensity of these two bands was expressed relatively to the intensity at the beginning of hydrolysis (Table 1). No significant changes of the band intensity occurred after 80 min incubation with Celluclast^®^ alone for the three cell types. Addition of the *Pa*LPMO9E to Celluclast^®^ resulted in almost 40% decrease in the intensity of the bands assigned to cellulose after 40 min incubation whereas lower changes were observed for parenchyma (30%) and tracheids (10%) suggesting structural important diffferences in the recalcitrance of chlorite-treated miscanthus cells. Such heterogeneity was still observed after 80 min incubation with *Pa*LPMO9E. Thus combining Celluclast^®^ and *Pa*LPMO9E during hydrolysis induced a sharper decrease in the absorption for both bands compared to Celluclast^®^ alone, especially for sclerenchyma. Faster and more severe degradation of cellulose was thus achieved on chlorite-treated miscanthus in the presence of *Pa*LPMO9E, and this enhancement depended on the cell type with sclerenchyma degradation being more affected compared to parenchyma. This result can be explained by the structural heterogeneity of corresponding cell walls (i.e. lignin content and composition, non-cellulosic matrix)^3,43^. Indeed delignification can impact on cellulose structure and removes some hemicelluloses and pectins. Interestingly, cellulose crystallinity was shown to increase after chlorite delignification^42^. One can suggest that altered crystallinity of cellulose in delignified sclerenchyma would explain the stronger boosting effect of *Pa*LPMO9E on enzymatic degradation of sclerenchyma.

**Table 1:** Change in FTIR absorbance at 1060 and 1105 cm^−1^ of chlorite-treated miscanthus vascular bundle area (sclerenchyma, parenchyma, tracheids) after 40 min and 80 min incubation with Celluclast^®^ alone or with *Pa*LPMO9E.

	**Sclerenchyma**		**Parenchyma**		**Tracheid**	
	40 min	80 min	40 min	80 min	40 min	80 min
**Celluclast** ^**®**^						
1060 cm^−1^	98 ± 2	93 ± 4	104 ± 1	109 ± 4	103 ± 3	99 ± 15
1105 cm^−1^	99 ± 1	94 ± 1	107 ± 1	118 ± 5	104 ± 4	111 ± 11
***Pa*** **LPMO9E + Celluclast** ^**®**^						
1060 cm^−1^	62 ± 3	39 ± 10	70 ± 2	65 ± 2	89 ± 4	76 ± 5
1105 cm^−1^	59 ± 1	40 ± 7	66 ± 11	60 ± 9	88 ± 1	78 ± 1

Band intensity values are expressed as percent of the intensity measured at the initial recording time. Starting recorded values (20 min) have been normalized at 100 for each wavenumber.

In conclusion, multimodal evaluation of the effect of *Pa*LPMO9E on enzymatic saccharification of miscanthus indicates a synergistic and boosting effect of *Pa*LPMO9E with cellulases on pretreated miscanthus. Experimental design setup at synchrotron allowed dynamic imaging of enzyme action working *in situ* under optimal conditions (temperature, hydrated substrate) and the corresponding changes in cell structure by real-time chemical monitoring. For the first time, the LPMO effect was found to depend on the tissue and the cell types in agreement with the heterogeneous structure and biological recalcitrance of cell walls. This approach could be extended to other bioenergy crops showing stem tissue heterogeneity like maize, wheat as well as deeper understanding of the synergic action of enzymes in lignocellulose saccharification. In addition real-time monitoring of enzyme action on lignocellulose using dedicated microscopic tools^29,46,47^ should provide complementary information at mesoscopic and molecular scales to improve efficiency of pretreatments and enzyme cocktail.

## Methods

### Lignocellulosic samples

Steam exploded miscanthus was provided by IFPEN (France). For synchrotron experiments, plant material was collected from the bottom stem region of *Miscanthus* x *giganteus* grown at INRA Estrées-Mons (France) and harvested at “dry” stage (February). Delignification pretreatment was performed on stem fragments (10 mm length × 3 mm thickness) using sodium chlorite-acetic acid as previously described^20^. Samples were washed several times with water until pH of the washings was neutral then embedded in polyethylene glycol to get serial 40 µm and 16 µm thickness sections for fluorescence imaging and FTIR microspectrometry respectively. Before using miscanthus samples, PEG was removed from the sections by washing with water (3 × 5 min). Another set of samples was dried into air forced oven at 40 °C and grinded prior to chemical analysis.

### Chemical analysis

Lignin content was quantified by spectroscopy using acetyl bromide. Polysaccharides composition was determined by quantifying sugar monomer released from sulfuric acid hydrolysis^20^.

### Saccharification assays

Saccharification assays were carried out on several pretreated miscanthus samples. *Pa*LPMO9E was produced and purified as previously described^32^. Supplementation assays were carried out in sodium acetate buffer (50 mM, pH 4.8) in a final volume of 1 mL at 0.5% consistency (weight dry matter per volume). Enzymatic treatments were performed in 2-mL tubes incubated at 50 °C and 850 rpm in a rotary shaker (Infors AG, Switzerland). *Pa*LPMO9E was added to the substrate at a concentration of 4 mg.g^−1^ dry matter in presence of 1 mM ascorbic acid for 72 hours, followed by addition of 12 filter paper units of commercial cellulases from Celluclast^®^ 1.5 L (Sigma-Aldrich), per gram of dry matter. After hydrolysis, samples were boiled for 10 min to stop the enzymatic reaction. After centrifugation (16,000 g, 5 min, 4 °C), glucose was quantified using the glucose RTU kit (Biomérieux, Marcy l′étoile, France), following the manufacturer’s instructions, and the DNS reagent using glucose calibration curve^48^. Assays were performed in triplicate. Oxidized cellobio-oligosaccharides were detected using HPAEC (high performance anionic-exchange chromatography chromatography)^28^. Previous study on the characterization of *Pa*LPMOE with different control reactions without enzyme indicated that ascorbate alone did not induce any modification of the substrate^32^.

For microscopic visualization of the dynamic changes at cell level, higher enzyme loadings (10 times more) were chosen to enable experiments within a reasonable timeframe of 2–3 hours. The reaction was performed in acetate buffer pH 4.8 (50 mM acetate containing 1 mM ascorbate) at 45 °C using the cellulase cocktail Celluclast^®^ 1.5 L, alone or in combination with *Pa*LPMO9E. Control experiments without enzymes were performed using the same buffer.

### Synchrotron fluorescence imaging

Multispectral image acquisition was achieved after a 280 nm excitation obtained from the DISCO beamline bending magnet at the SOLEIL synchrotron (Gif sur Yvette, France)^49^ using two filters: i) emission 327–353 nm to image enzyme (tryptophan autofluorescence)^45^, ii) 420–480 nm to image cell walls (phenolic components fluorescence). These conditions were selected by comparing images obtained at different excitation/emission filters. Miscanthus sections (40 µm thickness) were mounted between 2 quartz cover slides (160 µm in thickness) using a spacer to provide a 25 µL sealed chamber containing 22 µL enzymes–buffer solution (Fig. 3a). Reactions were performed at 45 °C using a microscope-adapted temperature-controlled stage (OkoLab, Italy). Time lapse images of the degradation were acquired sequentially at visible light and 275 nm and every 10 min during almost 2 hours for each experiment.

### Synchrotron FTIR microspectrometry

The data were acquired on the SMIS beamline in the SOLEIL synchrotron which exploits the edge and bending radiations from a bending magnet. The data were recorded on a Continuum XL microscope (ThermoScientifique, Courtaboeuf, France) equipped with a MCT-A detector. As for DISCO imaging, the incubation was performed at 45 °C thanks to the use of a temperature-controlled microfluidic device. Miscanthus sections (16 µm thickness) were mounted in a home-made microfluidic device between two ZnS windows using a 25 µm thickness ETFE spacer (Goodfellow, Huntingdon, UK) to make a chamber of 12 µL volume (Fig. 4a). Time lapse spectra were acquired every 5 min for 1 h then every 10 min for 1 h by selecting areas corresponding to different cell types. Spectra were recorded in transmission with 256 scans at 8 cm^−1^ resolution with a 12 × 12 µm² aperture. A background spectrum was recorded in water at the beginning of the experiment. The infrared region 1200–900 cm^−1^ was analyzed with baseline correction to monitor the extent of cell wall degradation. Peak intensities were measured at 1060 cm^−1^ and at 1107 cm^−1^ with a baseline between 1300 cm^−1^ and 950 cm^−1^.

### Statistical analysis

All experiments were performed as triplicates. Values in graphs and tables were presented as means with standard error of the means.

## Electronic supplementary material

SUPPLEMENTARY INFORMATION
