# Supplementary material for: Action of lytic polysaccharide monooxygenase on plant tissue is governed by cellular type
Source: Sci Rep. 2017 Dec 19;7:17792. doi: 10.1038/s41598-017-17938-2 (PMC5736606; doi:10.1038/s41598-017-17938-2)

Action of lytic polysaccharide monooxygenase on plant tissue is governed by cellular type

Brigitte Chabbert[1*], Anouck Habrant[1], Mickaël Herbaut[1], Laurence Foulon[1], Véronique Aguié-Béghin[1], Sona Garajova[2], Sacha Grisel[2], Chloé Bennati-Granier[2], Isabelle Gimbert-Herpoël[2], Frédéric Jamme[3], Matthieu Réfrégiers[3], Christophe Sandt[3], Jean-Guy Berrin[2] and Gabriel Paës[1]

[1] FARE Laboratory, INRA, University of Reims Champagne-Ardenne, 51100 Reims, France

[2] BBF, INRA, Aix Marseille University, Polytech'Marseille, 13288 Marseille, France

[3]Synchrotron SOLEIL, 91190 Saint Aubin, France

Figure S1: Effect of *Pa*LPMO9E on enzymatic hydrolysis of steam exploded miscanthus. (a) SEM imaging shows detructuration of steam exploded miscanthus (scale bar=300 µm); (b) *Pa*LPMO9E improved enzymatic degradation of miscanthus by Celluclast® ; (c) PaLPMO9E oxidation of steam exploded miscanthus releases C1-oxidized sugars as dimer (DP2C1-ox), trimer (DP3C1-ox), and tetramer (DP4C1-ox).

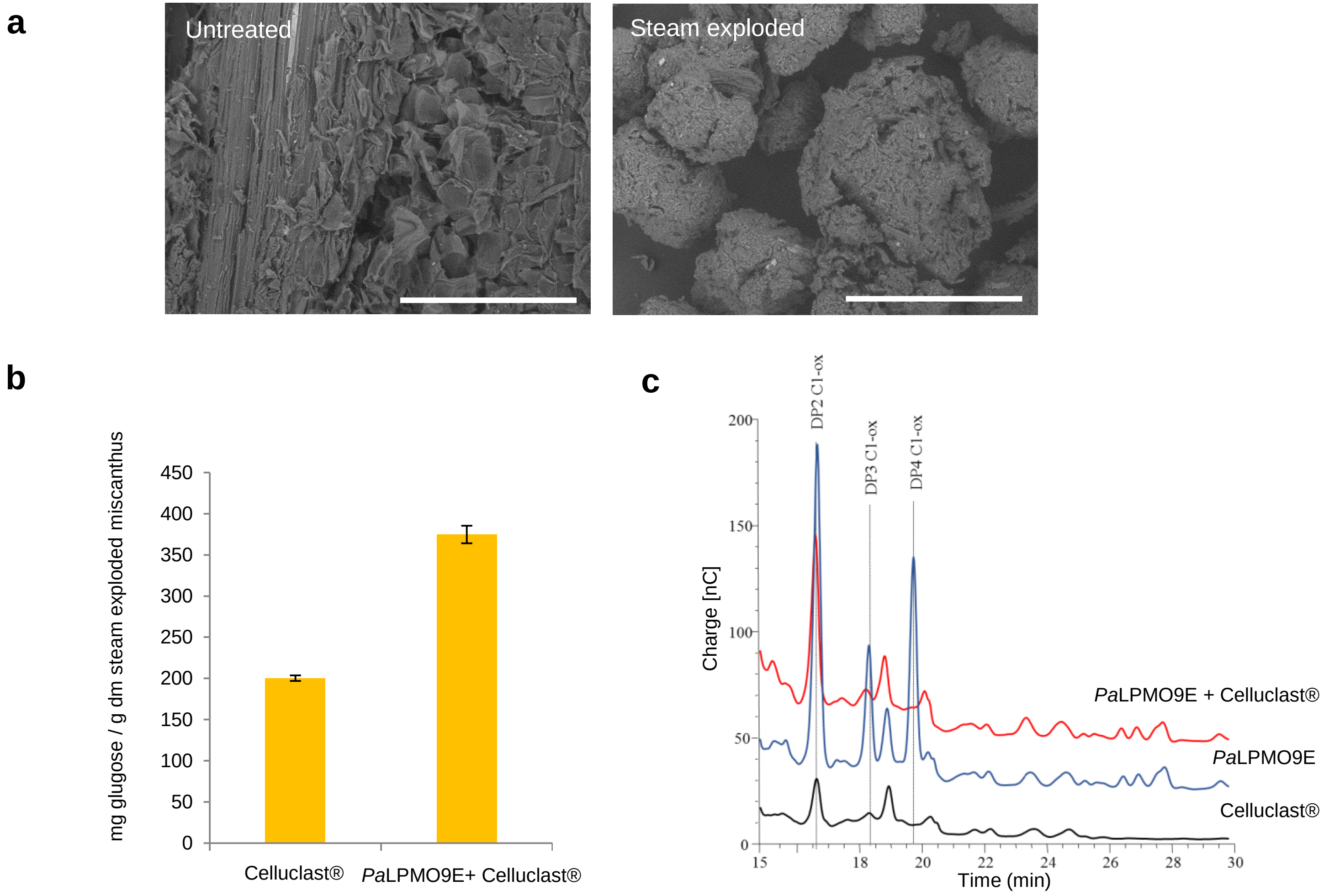

Figure S2: Chemical composition of untreated and chlorite-treated miscanthus. Lignin content is decreased by 5-fold, while relative content in arabinoxylan and cellulose increase.

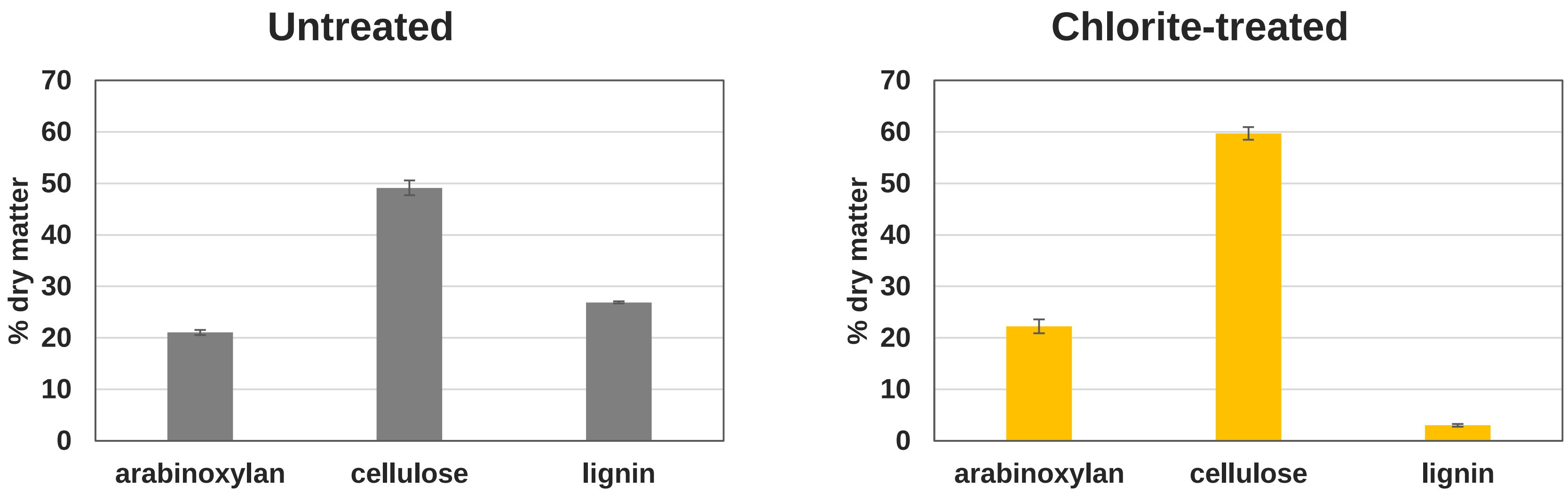

Figure S3: Time-lapse imaging of chlorite-treated miscanthus sample. Structure and fluorescence of region 1 are not modified over a time-period of more than 2 hours when incubated with *Pa*LPMO9E + Celluclast®. Scale bar = 200 µm.

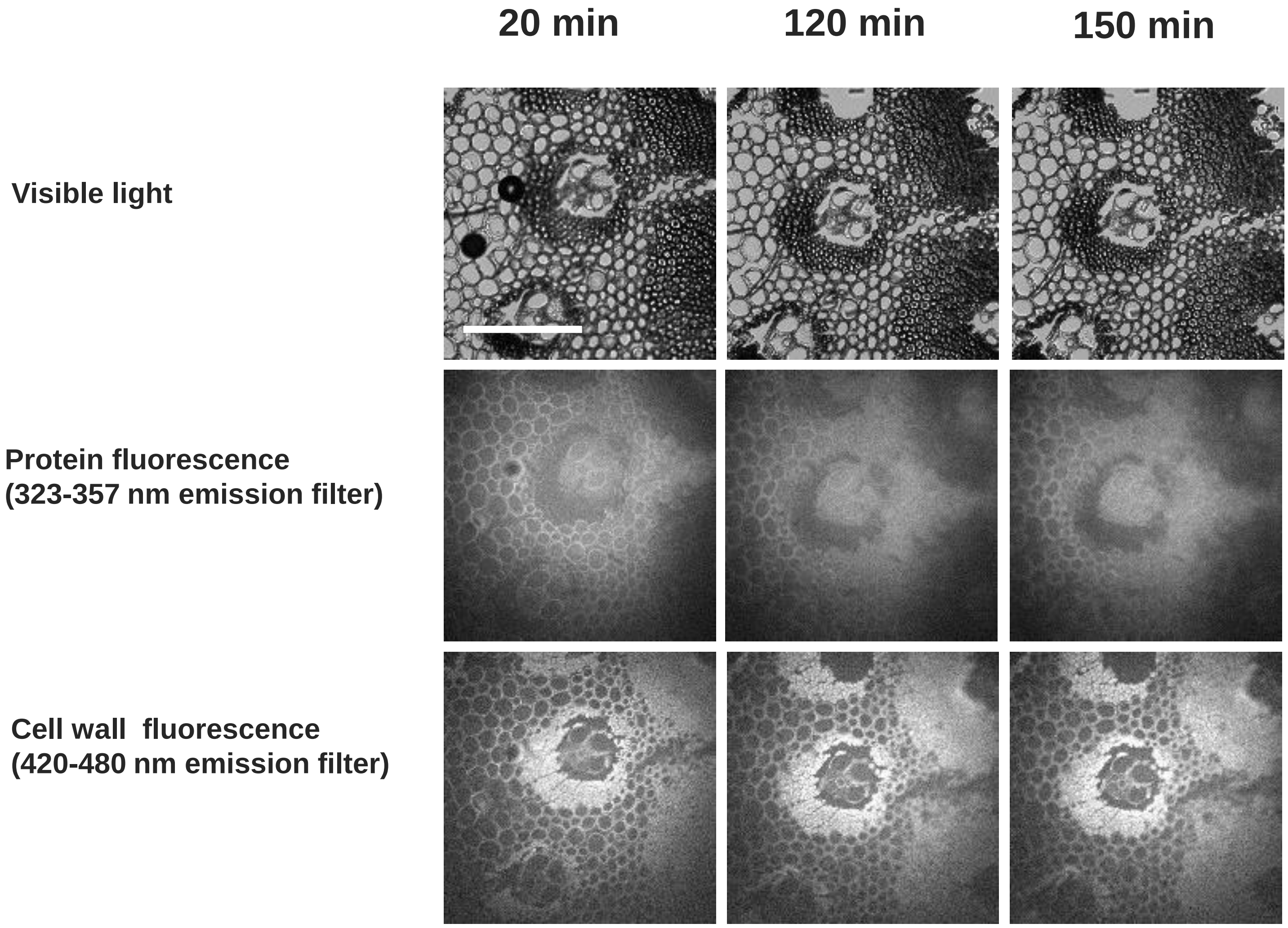

Figure S4: Time-lapse imaging of untreated miscanthus sample. Cell wall structure and fluorescence of region 2 are not modified over a time-period of more than 6 hours when incubated in the acetate buffer pH 4.5 at 45°C. Scale bar = 200 µm.

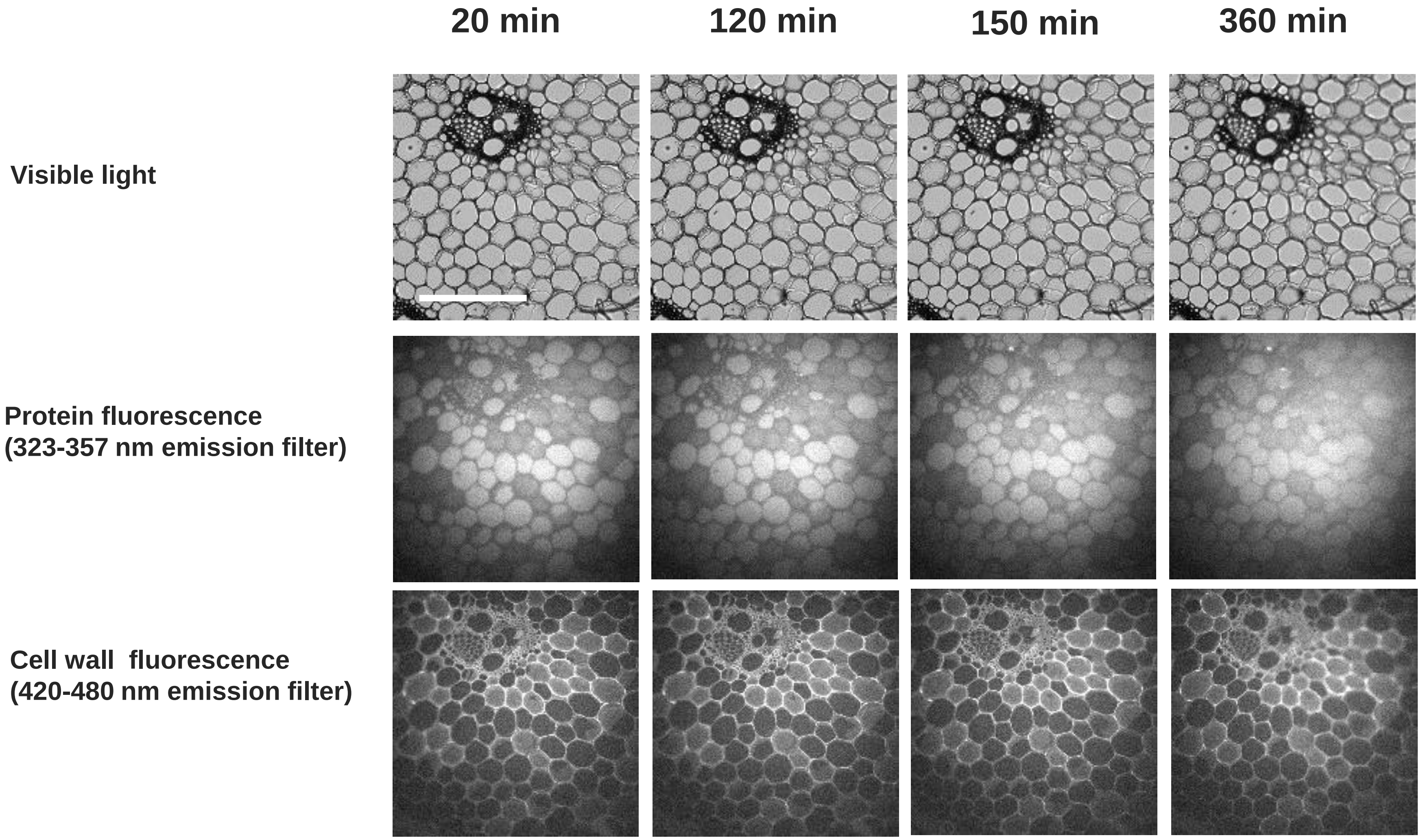

Figure S5: Time-lapse imaging of chlorite-treated miscanthus sample. Cell wall structure and fluorescence of region 2 are not modified over a time-period of more than 4 hours when incubated in the acetate buffer pH 4.5 at 45°C. Scale bar = 200 µm.

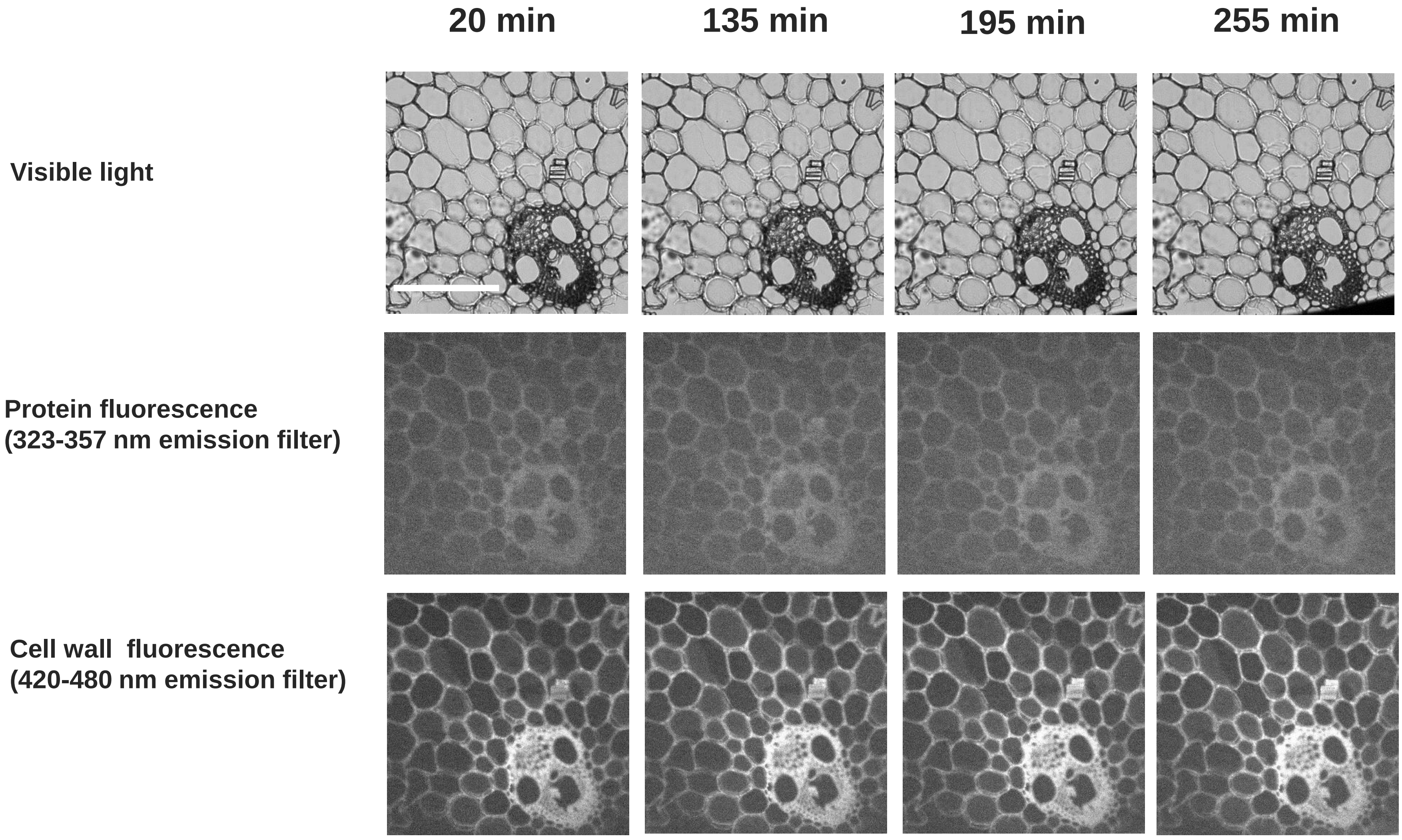

Figure S6: FTIR spectra of vascular bundle cells of untreated (a) and chlorite-treated (b) miscanthus after 20 min and 120 min incubation in buffer. Arrows indicate lignin band (1506 cm^{-1} and 1460 cm^{-1})

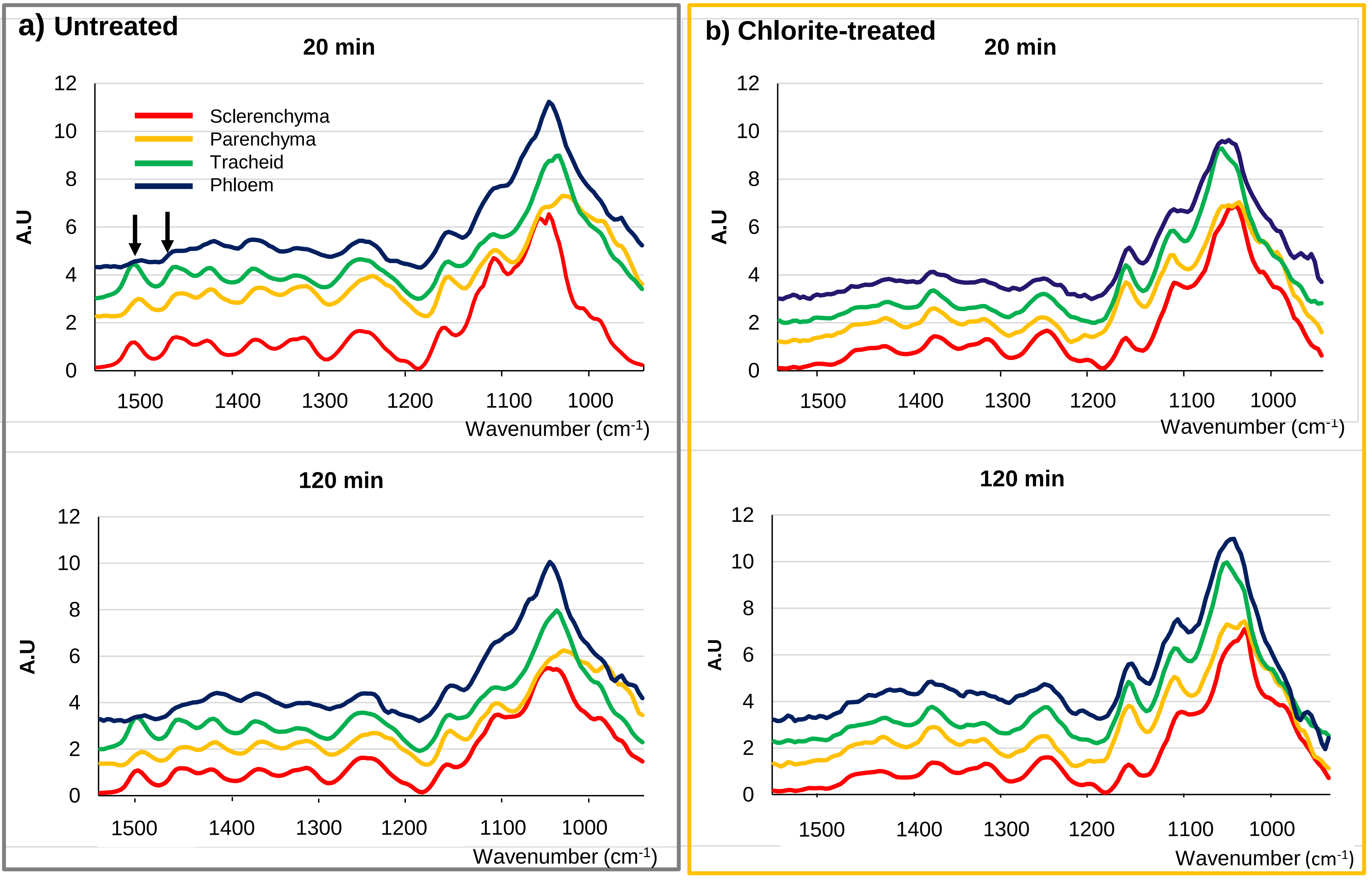

Supplement: Supplementary file 1 — SUPPLEMENTARY INFORMATION [file 41598_2017_17938_MOESM1_ESM.pdf]
